# BCG Skin Infection Triggers IL-1R-MyD88-Dependent Migration of EpCAM^low^ CD11b^high^ Skin Dendritic cells to Draining Lymph Node During CD4+ T-Cell Priming

**DOI:** 10.1371/journal.ppat.1005206

**Published:** 2015-10-06

**Authors:** Vishnu Priya Bollampalli, Lívia Harumi Yamashiro, Xiaogang Feng, Damiën Bierschenk, Yu Gao, Hans Blom, Birgitta Henriques-Normark, Susanne Nylén, Antonio Gigliotti Rothfuchs

**Affiliations:** 1 Department of Microbiology, Tumor and Cell Biology, Karolinska Institutet, Stockholm, Sweden; 2 Department of Pharmacology, Federal University of Santa Catarina, Florianópolis, Brazil; 3 Science for Life Laboratory, Royal Institute of Technology, Stockholm, Sweden; New Jersey Medical School, UNITED STATES

## Abstract

The transport of antigen from the periphery to the draining lymph node (DLN) is critical for T-cell priming but remains poorly studied during infection with *Mycobacterium bovis* Bacille Calmette-Guérin (BCG). To address this we employed a mouse model to track the traffic of Dendritic cells (DCs) and mycobacteria from the BCG inoculation site in the skin to the DLN. Detection of BCG in the DLN was concomitant with the priming of antigen-specific CD4^+^ T cells at that site. We found EpCAM^low^ CD11b^high^ migratory skin DCs to be mobilized during the transport of BCG to the DLN. Migratory skin DCs distributed to the T-cell area of the LN, co-localized with BCG and were found in close apposition to antigen-specific CD4^+^ T cells. Consequently, blockade of skin DC traffic into DLN dramatically reduced mycobacterial entry into DLN and muted T-cell priming. Interestingly, DC and mycobacterial entry into the DLN was dependent on IL-1R-I, MyD88, TNFR-I and IL-12p40. In addition, we found using DC adoptive transfers that the requirement for MyD88 in BCG-triggered migration was not restricted to the migrating DC itself and that hematopoietic expression of MyD88 was needed in part for full-fledged migration. Our observations thus identify a population of DCs that contribute towards the priming of CD4^+^ T cells to BCG infection by transporting bacilli into the DLN in an IL-1R-MyD88-dependent manner and reveal both DC-intrinsic and -extrinsic requirements for MyD88 in DC migration.

## Author Summary

The arrival of bacilli in the lymph node is a bottleneck for initiating T cell responses to mycobacteria but remains poorly studied. To address this we used a mouse model to track the entry of cells and bacteria into the lymph node during skin infection with *Mycobacterium bovis* BCG, the live tuberculosis vaccine. We identified a population of migratory Dendritic cells that transport bacilli from the skin into the lymph node and which engage CD4^+^ T cells therein. The mobilization of Dendritic cells from the skin, and with these cells the transport of mycobacteria into the lymph node, was regulated by cytokines, in particular Interleukin–1. This was also dependent on MyD88, an adaptor molecule downstream of the Interleukin–1 receptor. We also found a requirement for MyD88 in driving Dendritic cells to the lymph node that was both inherent and extrinsic to the migrating cell. These findings bare consequences for our understanding of how T-cell responses are initiated during microbial challenge in the skin and hold promises for improving vaccines of low-to-modest efficacy such as BCG, which rely on such responses.

## Introduction

Lymph nodes (LNs) make use of lymphatic drainage and a specialized microanatomy to facilitate productive encounters between antigen-laden Dendritic cells (DCs) and naïve T cells [1]. As sentinel phagocytes that reconnoiter for infection, DCs employ an array of pattern-recognition receptors (PRRs) to sense microbes or their metabolites [2]. Microbial triggering of PRRs unleashes an intracellular signaling cascade in DCs that culminates in enhanced antigen presentation, up-regulation of co-stimulatory molecules and cytokine production. This activation process enables DCs upon engaging a naïve T cell clone to direct the expansion and differentiation of that clone into an armed, effector T-cell population [3]. These dynamic cellular interactions that unfold in the LN mark the initiation of cell-mediated immunity that are critical for host resistance to infection.

*Mycobacterium tuberculosis*, the etiological agent of tuberculosis, is second only to HIV/AIDS as the largest cause of death in the World due to a single microorganism [4]. Host resistance to mycobacteria relies heavily on cell-mediated immunity and IFN-γ [5,6]. Mounting a robust immune response mediated by Th1 effector cells is thus an important outcome for successful tuberculosis vaccination. In this context, cutaneous infection with the attenuated strain of *M*. *bovis* called Bacille Calmette-Guérin (BCG) is the only vaccination available against *M*. *tuberculosis*, but is lacking in clinical efficacy. Improving BCG is a valid strategy to promote global control of tuberculosis [7] and understanding how CD4^+^ T cells are primed by BCG *in situ* sets a rational basis towards the latter. However, there is a paucity of information regarding the initial steps that ensue upon BCG infection and which culminate in the generation of a Th1 response. In particular, the channeling of antigen from the BCG-inoculation site in the skin to the draining LN (DLN) remains poorly studied.

A large body of data implicate DCs in the active transport of antigen from the periphery to the DLN [1] but the identification of several DC sub-populations in the skin [8] has added to the complexity of this event. Although molecular mechanisms of motility have been investigated in several studies [9,10], the *in vivo* contribution of PRRs, their signaling pathways and cytokines await full elucidation during DC migration triggered by infection. Here we developed a method to track the movement of cells and mycobacteria from the footpad to the popliteal DLN to study this during BCG infection. We found that migratory EpCAM^low^ CD11b^high^ skin DCs were the main DC sub-population mobilized during the transport of BCG to the DLN. This process, associated with the priming of antigen-specific CD4^+^ T cells, was dependent on Interleukin–1 receptor (IL-1R) and the Toll-like receptor (TLR)/IL-1R adaptor molecule MyD88. In addition, MyD88 played both a DC-intrinsic and -extrinsic role in BCG-triggered migration.

## Results

### Priming of BCG-specific CD4^+^ T cells is concentrated to the DLN

**Fig 1:**
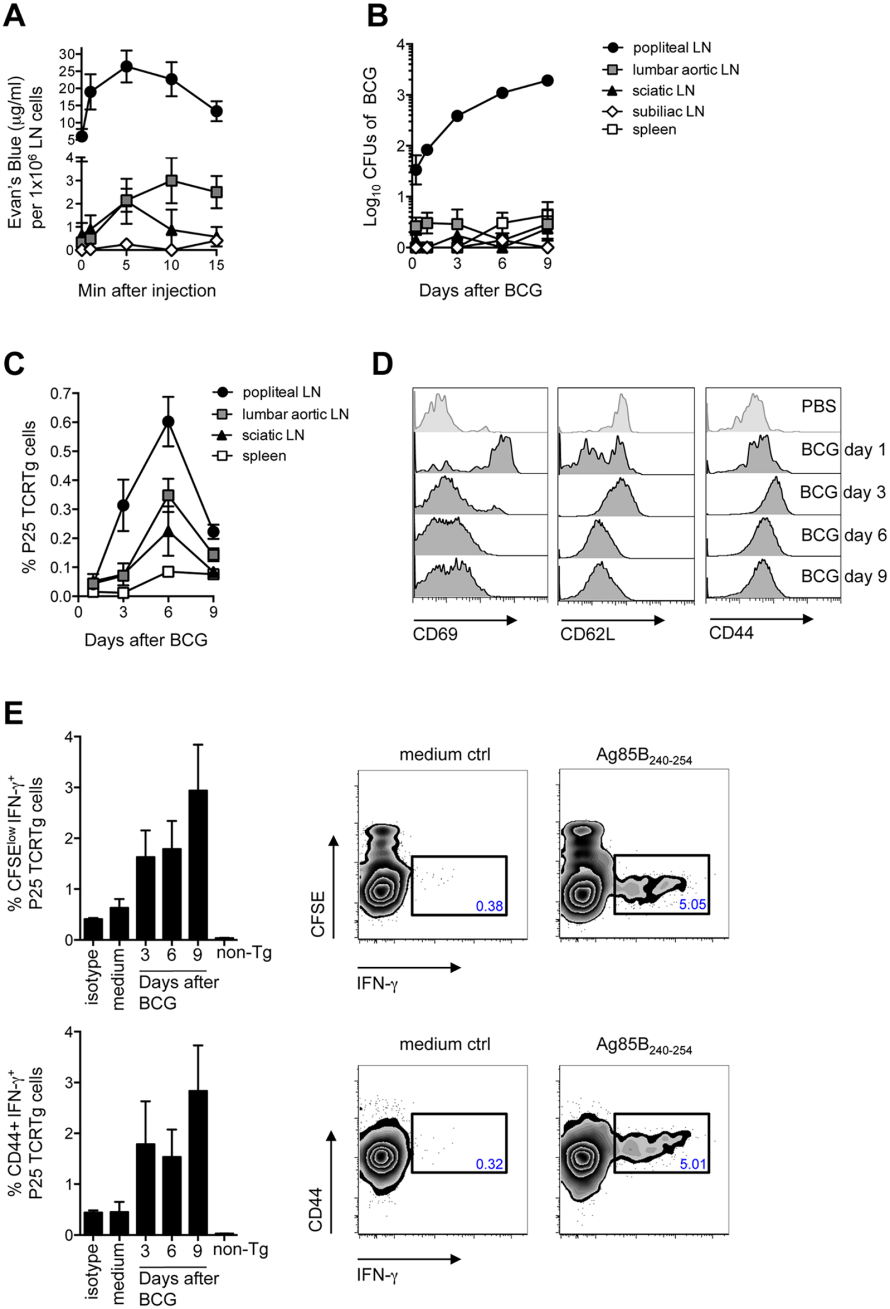
Detection of BCG in the DLN correlates with the priming of P25 TCRTg cells. (**A**) 5% Evan’s blue was injected in the footpad of WT mice. At the indicated time points, animals were sacrificed, LNs isolated and the amount of dye per LN determined on a spectrophotometer against a standard curve. The concentration of Evan’s blue was normalized to the average cell number in each LN. (**B**) WT mice were inoculated with 1x10^6^ CFUs of BCG in the footpad. CFUs of BCG in LNs and spleen were enumerated on 7H11 agar at the indicated time points after infection. (**C**) Frequency of P25 TCRTg cells was determined by flow cytometry in LNs and spleen of BCG-infected mice at the indicated time points after infection. Naïve P25 TCRTg cells were CFSE-labeled and transferred into CD45.1^+^ recipient mice inoculated 24hrs later with BCG as in (**B**). (**D**) Changes in MFI for CD69 (left panel), CD62L (center panel) and CD44 (right panel) on transferred P25 TCRTg cells (CD4^+^ CD45.2^+^) in the pLN at different time points after BCG infection. (**E**) Intracellular production of IFN-γ on transferred P25 TCRTg cells from pLN after *in vitro* recall with Ag85B_240–254_ peptide. Zebra plots depict intracellular IFN-γ in P25 TCRTg cells 9 days after BCG infection, following recall with peptide. Only baseline levels of IFN-γ were observed on non-transgenic (non-Tg) LN cells (CD45.2^neg^ CD4^+^), shown here on day 9 after infection. Four to 8 animals per group were used in each experiment. Bars indicate standard error of the mean. One of two independent experiments shown.

To begin dissecting the early events following BCG infection, we established a model where C57BL/6 mice are inoculated with BCG in the footpad skin and immune responses assessed in the DLN. The popliteal LN (pLN) was established as the DLN after injection of Evan’s Blue in the footpad, with secondary drainage to lumbar aortic and sciatic LNs (Fig 1A). In accordance with the above, inoculation of BCG in the footpad lead to a major detection of bacilli in the draining, pLN (Fig 1B). To examine the outcome of mycobacterial antigen-specific CD4^+^ T-cell responses in this setting, the fate of naïve P25 TCRTg cells was followed *in vivo*. These CD4^+^ T cells have a transgenic T-cell receptor specific for peptide 25 of mycobacterial antigen 85B (Ag85B_240–254_) [11]. The expansion of P25 TCRTg cells was first evident in the pLN, which was also the dominant site for P25 TCRTg cell expansion (Fig 1C). Up-regulation of CD69 and down-regulation of CD62L were clearly evident on P25 TCRTg cells 1 day after BCG infection, prior to T-cell expansion (Fig 1D). The expansion of P25 TCRTg cells correlated with sequential dilution of CFSE labeling in this population and was corroborated by an increase in the absolute number of activated P25 TCRTg cells in the DLN (S1A and S1C Fig). As reported by others, [12], surface expression of CD69 on transgenic cells was progressively down-regulated with cell division while cells that had divided expressed more CD44 compared to undivided cells (S1B Fig). Moreover, P25 TCRTg cells were found to produce IFN-γ upon recall with Ag85B_240–254_. IFN-γ^+^ cells were CFSE^low^ and CD44^+^ (Fig 1E). Overall, our observations suggest that the priming of P25 TCRTg cells is concentrated to the DLN where also the majority of culturable bacilli are found.

### EpCAM^low^ CD11b^high^ DCs are the main DC population relocating from the skin to the DLN after BCG infection

**Fig 2:**
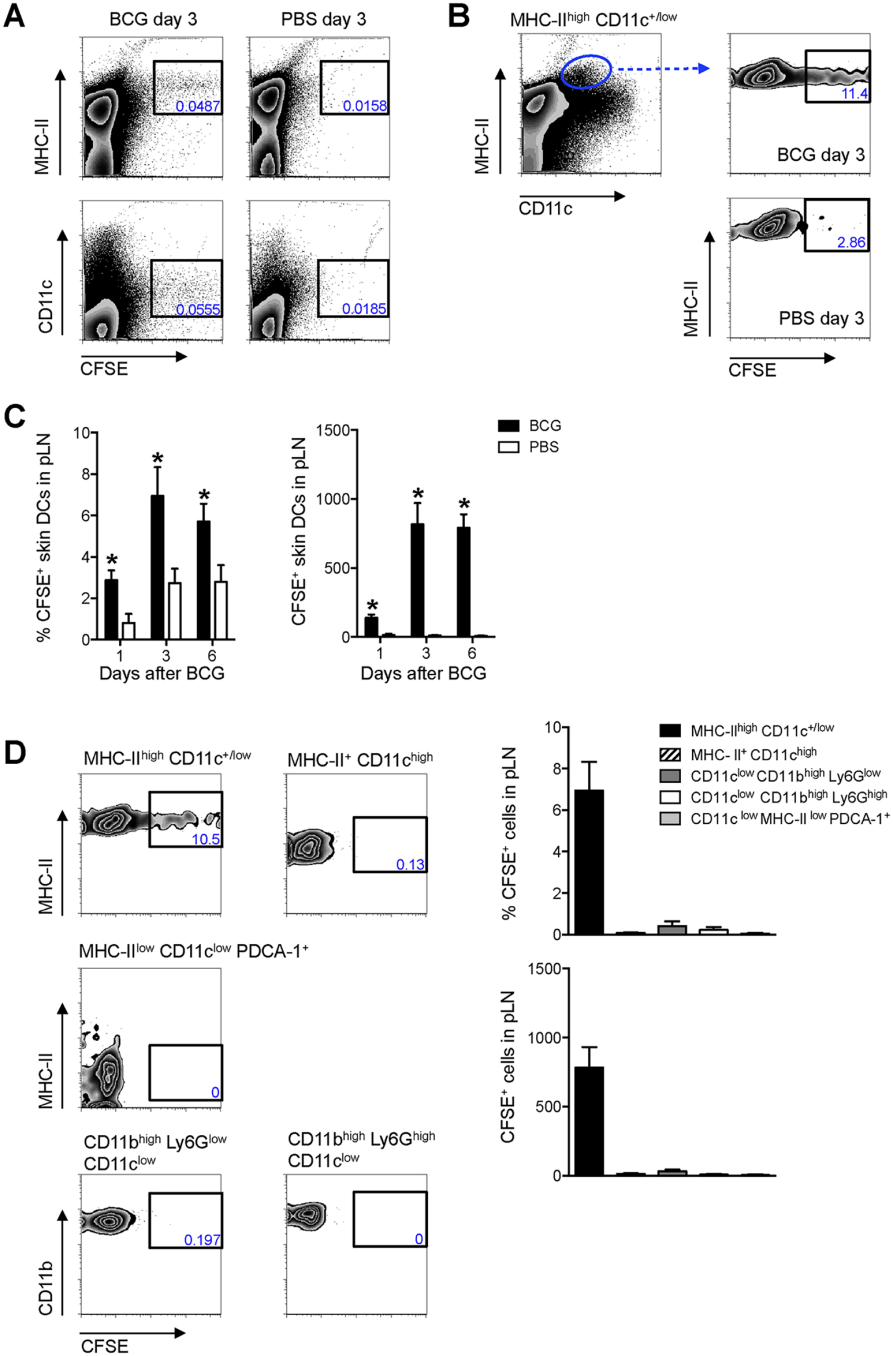
Migratory skin DCs are a major population relocating to the DLN after BCG footpad infection. WT mice were infected with BCG in the footpad as in Fig 1B. Twenty-four hrs before sacrifice, animals were injected with 0.5 mM CFSE in the same footpad. Popliteal LNs were harvested, homogenized into single-cell suspensions and subjected to flow cytometry. For measurements made 1 day after BCG, CFSE was injected 2hrs after the inoculation of BCG. (**A** and **B**) Zebra plots showing predominant expression of CFSE on MHC-II^high^ and CD11c^+/low^ cells in BCG-draining pLN 3 days after infection. (**C**) Frequency and total number of CFSE-labeled MHC-II^high^ CD11c^+/low^ cells in pLN from BCG- and PBS-injected mice were graphed at different time points. (**D**) CFSE expression within different myeloid cell populations was determined by flow cytometry 3 days after BCG: migratory skin DCs (MHC-II^high^ CD11c^+/low^), LN-resident DCs (MHC-II^+^ CD11c^high^), plasmacytoid DCs (MHC-II^low^ CD11c^low^ PDCA–1^+^), neutrophils (CD11c^low^ CD11b^high^ Ly6G^high^), and monocyte/ macrophages (CD11c^low^ CD11b^high^ Ly6G^low^). Gating strategies for these populations are presented in S3 Fig. Frequency and total number of CFSE^+^ cells present within each population was graphed. For each experiment, at least 5 mice were used for BCG-infected groups and 3 for PBS-injected controls. Bars indicate standard error of the mean. *Denotes statistically significant differences between BCG-infected and PBS controls. One of two independent experiments shown.

The above did not establish whether active cell migration from the site of BCG inoculation in the skin coincided with T-cell priming in the DLN. A migration assay was thus developed to address this. CFSE was directly injected into the same footpad previously inoculated with BCG and the draining, pLN analyzed 24hrs later for CFSE-labeled cells using flow cytometry. Given their central role in T-cell priming, we focused our investigations on DCs. Indeed, a large portion of CFSE labeling in the pLN was found on MHC-II^high^ and CD11c^+/low^ cells (Fig 2A), consistent with skin DCs that have migrated to DLN [8]. Both the frequency and total numbers of CFSE-labeled, MHC-II^high^ CD11c^+/low^ cells were increased in BCG-infected animals as compared to PBS-injected controls (Fig 2B and 2C), indicating that BCG prompts mobilization of these cells to the DLN. CFSE-labeled migratory skin DCs had increased expression of CD80 and CD86 regardless if isolated from BCG- or PBS-injected animals (S2 Fig). This suggests that skin DCs arriving into the DLN during the 24hr period of our migration assay constitute an activated cell population, consistent with previous findings from skin explant cultures and FITC skin painting [13]. Importantly, LN-resident DCs (MHC-II^+^ CD11c^high^ cells) where negative for CFSE (Fig 2D), suggesting that CFSE labeling occurred primarily in the footpad, as expected. In line, plasmacytoid DCs (MHC-II^low^ CD11c^low^ PDCA–1^+^ cells), known to enter inflamed LNs through high endothelial venules (HEVs) [14], were also negative for CFSE, as were monocytes (CD11c^low^ CD11b^high^ Ly6G^low^ cells) (Fig 2D). Contrary to a previous report [15], BCG did not trigger the migration of neutrophils (CD11c^low^ CD11b^high^ Ly6G^high^ cells) from the skin to the DLN (Fig 2D), suggesting that in our particular model, skin DCs predominate over neutrophils in this regard.

**Fig 3:**
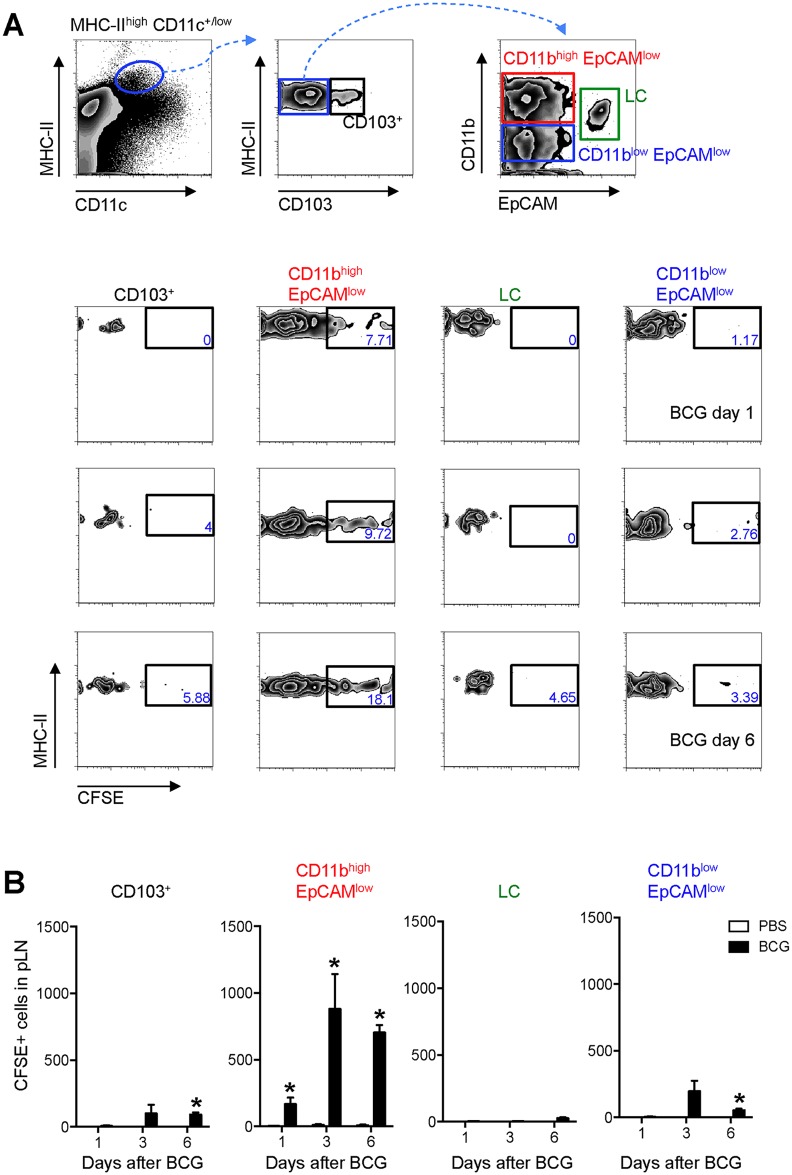
EpCAM^low^ CD11b^high^ DCs are the main skin DC subset trafficking to the DLN after BCG infection. Frequency (**A**) and total number (**B**) of CFSE^+^ cells within defined subsets of migratory skin DCs in pLN after BCG infection. (**A**) Zebra plots showing gating strategy employed (top panel) and the frequency of CFSE^+^ cells within each, defined subset at different time points after BCG infection (bottom panels). The frequency of CFSE^+^ cells within the EpCAMl^ow^ CD11b^high^ population in BCG-draining pLN is statistically significant relative to PBS-draining pLN for all three time points analyzed. (**B**) Total number of CFSE^+^ cells within each subset as defined in (**A**). For each experiment, at least 5 mice were used for BCG-infected groups and 3 for PBS controls. Bars indicate standard error of the mean. *Denotes statistically significant differences relative to PBS-injected controls. One of three independent experiments shown.

Since MHC-II^high^ CD11c^+/low^ cells in skin DLNs encompass multiple DC sub-populations [8], this subset was further characterized using CD103, EpCAM (CD326), and CD11b, markers previously employed in combination to define subsets of migratory skin DCs [16–19]. In this manner, a sub-population of EpCAM^low^ CD11b^high^ cells was identified as the main migratory DC subset in response to BCG infection in our model (Fig 3).

### CFSE^+^ cells contain BCG and localize to the T-cell area of the LN

**Fig 4:**
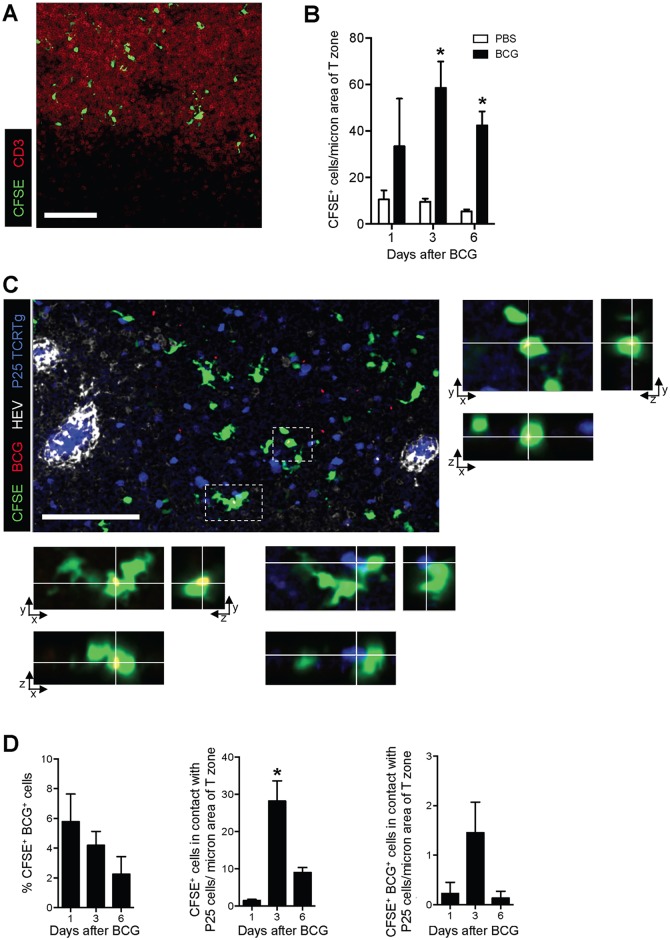
CFSE^+^ cells enter the paracortex, co-localize with BCG and are closely apposed to P25 TCRTg cells. P25 TCRTg cells were isolated from LNs of naïve P25 TCRTg RAG–1^-/-^ ECFP mice using a CD4 negative selection procedure (Miltenyi Biotec), and transferred i.v. into WT recipients. Recipients were then inoculated with BCG-RFP and the CFSE-based migration assay performed. (**A**) Distribution of CFSE^+^ cells in the paracortex (T-cell area) of the pLN as determined by CD3 staining on pLN sections from BCG-injected mice. (**B**) Quantification of CFSE^+^ cells within the T-cell area of the pLN from BCG- and PBS-injected mice expressed as the number of CFSE^+^ cells per area of CD3 staining. (**C**) Micrograph showing distribution of BCG (red), CFSE^+^ cells (green), p25 TCRTg cells (blue) and HEVs (white) in the pLN. Boxed inserts in the micrograph were magnified to show x/y/z projections of two CFSE^+^ cells with intracellular BCG-RFP. BCG were overexposed in these magnified views to facilitate visualization. Infected cell in lower panel is also in contact with a p25TCRTg ECFP cell (blue). (**D**) Quantification of the number of CFSE^+^ cells containing BCG-RFP (left graph), found in close apposition with P25 TCRTg ECFP cells (center graph), and containing BCG-RFP and in close apposition with P25 TCRTg ECFP cells (right graph). Data samples represent average obtained from individual pLN from which multiple fields of maximum intensity projections were analyzed. Bars indicate standard error of the mean. White bar on micrographs depict 100 microns. *Denotes statistically significant difference between BCG-infected and PBS controls in (**B**) and between day 1 and 6 in (**D**).

Next, the distribution of these migratory skin DCs was assessed in the DLN. The CFSE-based migration assay was performed and sections of pLN from PBS- and BCG-infected animals were subjected to confocal microscopy. CFSE-labeled cells localized exclusively to the T-cell area of the LN (Fig 4A) and in increased numbers following BCG but not PBS injection (Fig 4B), the latter corroborating our flow cytometry data. The presence of BCG within this migratory cell-population was confirmed at all time-points analyzed by using DsRed-expressing bacilli (BCG-RFP) [15] (Fig 4C and 4D). To test whether migrating skin DCs were capable of engaging BCG-specific CD4^+^ T cells, naïve P25 TCRTg cells expressing ECFP were adoptively transferred into recipient mice. The latter were then infected with BCG-RFP in the footpad and the CFSE-based migration assay performed. Several CFSE^+^ cells were found in apposition to P25 TCRTg cells (Fig 4C and 4D), suggesting a possible role for this migratory DC sub-population in priming CD4^+^ T cells to BCG. Interestingly, such positioning was independent of whether or not CFSE^+^ cells were directly infected with BCG.

### Inhibition of skin DC migration reduces BCG load in DLN and mutes CD4^+^ T-cell priming

**Fig 5:**
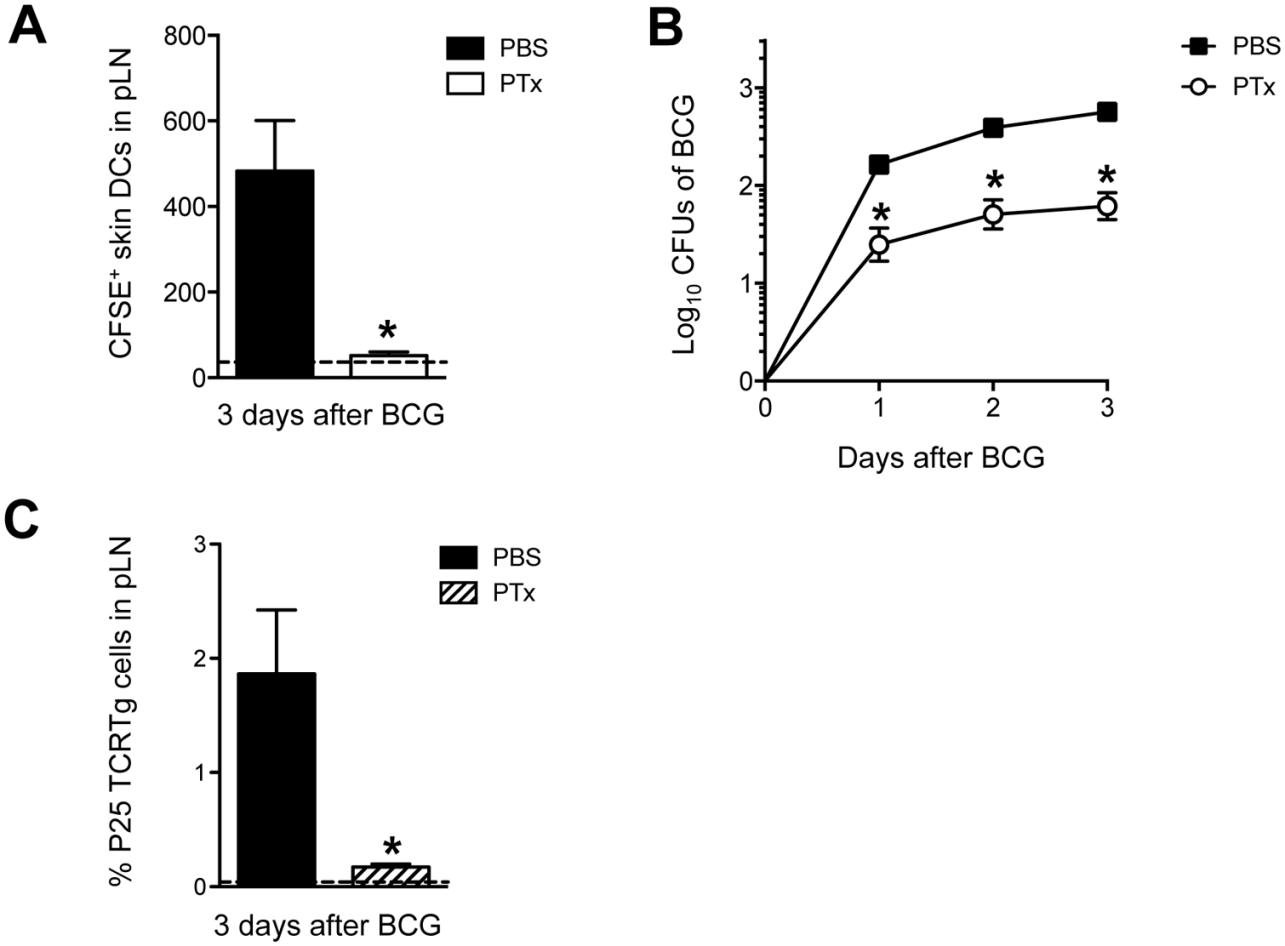
PTx mutes P25 TCRTg-cell priming by inhibiting skin DC migration and BCG transport to DLN. (**A**) Total number of CFSE^+^ MHC-II^high^ CD11c^+/low^ skin DCs in BCG-draining pLN from animals treated in the footpad with PTx. WT mice received an injection of 1 μg PTx (Sigma) or PBS in the footpad. Animals were inoculated 4hrs later with BCG in the same footpad. CFSE was injected into the same footpads 24hrs before sacrifice. Three days after BCG, pLNs were isolated and frequency of migrating skin DCs analyzed by flow cytometry. Dashed line depicts average number of CFSE^+^ skin DCs in uninfected, PBS-injected controls. (**B**) CFUs of BCG in pLN at the indicated time points after infection were determined on 7H11 agar from WT mice treated with PTx or PBS as in (**A**). (**C**) Naïve P25 TCRTg cells were CFSE-labeled and transferred into congenic CD45.1^+^ recipients. Twenty-four hrs later, animals were treated with PTx as in (**A**) and inoculated with BCG in the same footpads. Expansion of P25 TCRTg cells was determined 3 days later by flow cytometry. Dashed line depicts average frequency of P25 TCRTg cells obtained from uninfected, PBS-injected controls. For each experiment, at least 5 mice were used for BCG-infected groups and 3 for PBS controls. Bars indicate standard error of the mean. One of two independent experiments shown. *Denotes statistically significant difference in BCG-infected, PTx-treated group compared to BCG-infected, PBS-treated animals.

To further study the contribution of migratory skin DCs in T-cell priming, Pertussis toxin (PTx) was injected in the footpad of mice prior to BCG infection as a means of blocking DC migration [20]. Such treatment with PTx lead to a total ablation of BCG-triggered skin DC mobilization to DLN (Fig 5A) and a dramatic reduction in the number of BCG Colony-forming units (CFUs) in the DLN (Fig 5B). Importantly, PTx treatment muted the early expansion of naïve P25 TCRTg cells in the DLN (Fig 5C). PTx did not seem to have a major, direct impact on P25 TCRTg cells as the activation profile of this population in PTx-treated, BCG-infected animals was similar to that of infected controls (S4A and S4B Fig). Similarly, injection of PTx 3 days after BCG infection, when skin DCs and mycobacteria had already reached the DLN, did not alter the activation of naïve P25 TCRTg cells transferred at the time of PTx treatment (S4C Fig). A direct effect of PTx on BCG growth was also ruled out since addition of PTx to BCG cultures did not affect mycobacterial growth in 7H11 agar (S4D Fig). Thus, upon localized injection in the footpad, PTx seems mainly to inhibit skin DC migration to DLN and consequently, the entry of BCG to the DLN. These observations support a role for migratory skin DCs in channeling BCG to the DLN and in doing so, the priming of CD4^+^ T cells is unleashed therein.

### Skin DC migration and BCG entry into DLN is regulated by IL-1R, MyD88, IL-12p40 and TNFR-I

**Fig 6:**
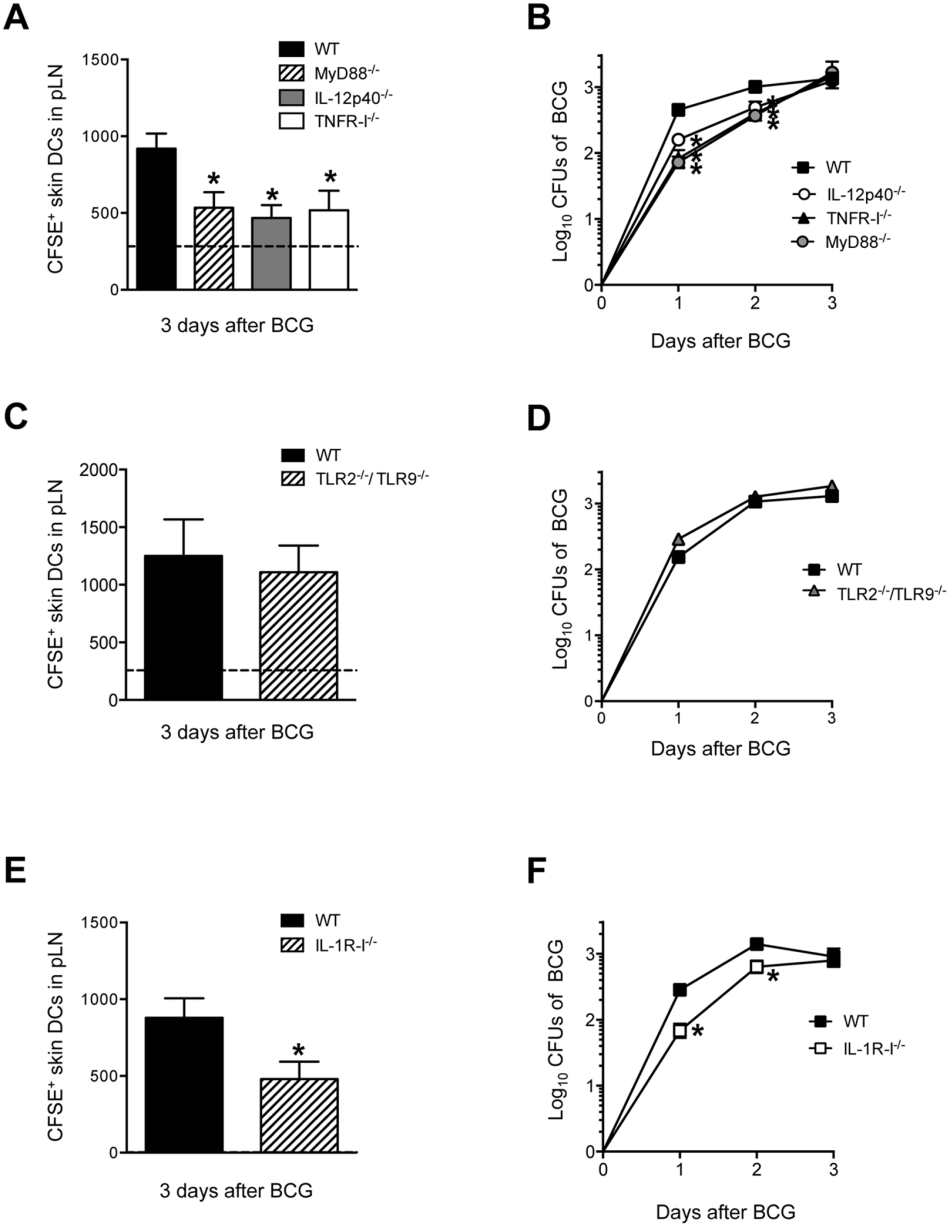
MyD88, IL-12p40, TNFR-I and IL-1R-I regulate entry of skin DCs and BCG into DLN. (**A**, **C** and **E**) Total number of CFSE^+^ MHC-II^high^ CD11c^+/low^ skin DCs in BCG-draining pLN of WT, MyD88^-/-^, IL-12p40^-/-^, TNFR-I^-/-^ (**A**), WT and TLR2^-/-^/TLR9^-/-^ (**C**) and WT and IL-1R-I^-/-^ mice (**E**) 3 days after BCG footpad infection. BCG and CFSE were injected as in Fig 2 and CFSE^+^ cells analyzed by flow cytometry. Dashed line depicts average number of CFSE^+^ MHC-II^high^ CD11c^+/low^ skin DCs in PBS-injected WT controls. Due to large experimental groups, data from TNFR-I^-/-^ mice were pooled from a separate cohort. (**B**, **D** and **F**) Bacterial load in pLN was determined after BCG footpad infection in WT, MyD88^-/-^, IL-12p40^-/-^, TNFR-I^-/-^ (**B**), WT and TLR2^-/-^/TLR9^-/-^ (**D**), and WT and IL-1R-I^-/-^ (**F**) mice at the indicated time points after infection. Five to 10 animals per time point and group depicted in BCG-infected cohorts, and 3 to 6 animals for PBS-injected controls. One of 5 independent experiments for WT, MyD88^-/-^, IL-12p40^-/-^ mice, one of two independent experiments for TNFR-I^-/-^, IL-1R-I^-/-^ and TLR2^-/-^/TLR9^-/-^ mice. Bars indicate standard error of the mean. *Denotes statistically significant differences between WT and gene-targeted mice.

Next, the mechanism behind BCG-triggered skin DC migration to DLN was studied. Given the prominent role of the IL-1R/TLR adaptor molecule MyD88 in the activation of antigen-presenting cells and the initiation of Th1 responses to mycobacteria [21,22], we decided to investigate this molecule in our model by using MyD88^-/-^ mice. Indeed, MyD88^-/-^ mice revealed a major reduction in BCG-triggered skin DC migration to pLN as compared to WT controls (Fig 6A). MyD88^-/-^ mice also displayed an important reduction in BCG CFUs in the pLN compared to WT controls (Fig 6B), providing additional support for skin DCs in the transport of BCG to the DLN. Since mycobacteria-triggered DC production of IL-12p40 and TNF-α are MyD88-dependent in murine DC cultures [22–24], we also decided to investigate the role of these cytokines in migration. Interestingly, a deficit in DC mobilization and BCG entry into the DLN was observed in IL-12p40^-/-^ and TNFR-I^-/-^ mice (Fig 6A and 6B). These findings corroborate the well-established role of TNF-α as a trigger of skin DC migration [25] and support intriguing but isolated reports that IL-12p40 homodimer regulates DC migration during bacterial infection [26–28]. However, TLR2 and TLR9, two of the main TLRs engaged in mycobacterial sensing by DCs, were not required for skin DC mobilization or BCG transport to DLN (Fig 6C and 6D). On the other hand, IL-1R-I^-/-^ mice displayed a reduction in BCG-triggered skin DC migration and BCG CFUs in the DLN (Fig 6E and 6F) similar to that of MyD88^-/-^ mice, suggesting that the DC and BCG relocation defects in the DLN of MyD88^-/-^ mice reflect a defect in IL-1R rather than TLR signaling. In line with MyD88 and IL-1R-I being important for skin DC migration and BCG entry into DLN, we found that the early activation of naïve P25 TCRTg cells transferred into MyD88^-/-^ and IL-1R-I^-/-^ recipients was reduced compared to P25 TCRTg cells that were transferred into BCG-infected WT controls (S5 Fig). Furthermore, it was also observed that administration of IL-12p40 homodimer but not TNF-α to footpads restores BCG-triggered skin DC migration in IL-1R-I^-/-^ mice (S6 Fig), suggesting that the ability of TNF-α to regulate migration in our model may be dependent on IL-1R.

### DC migration requires DC-intrinsic and -extrinsic expression of MyD88

**Fig 7:**
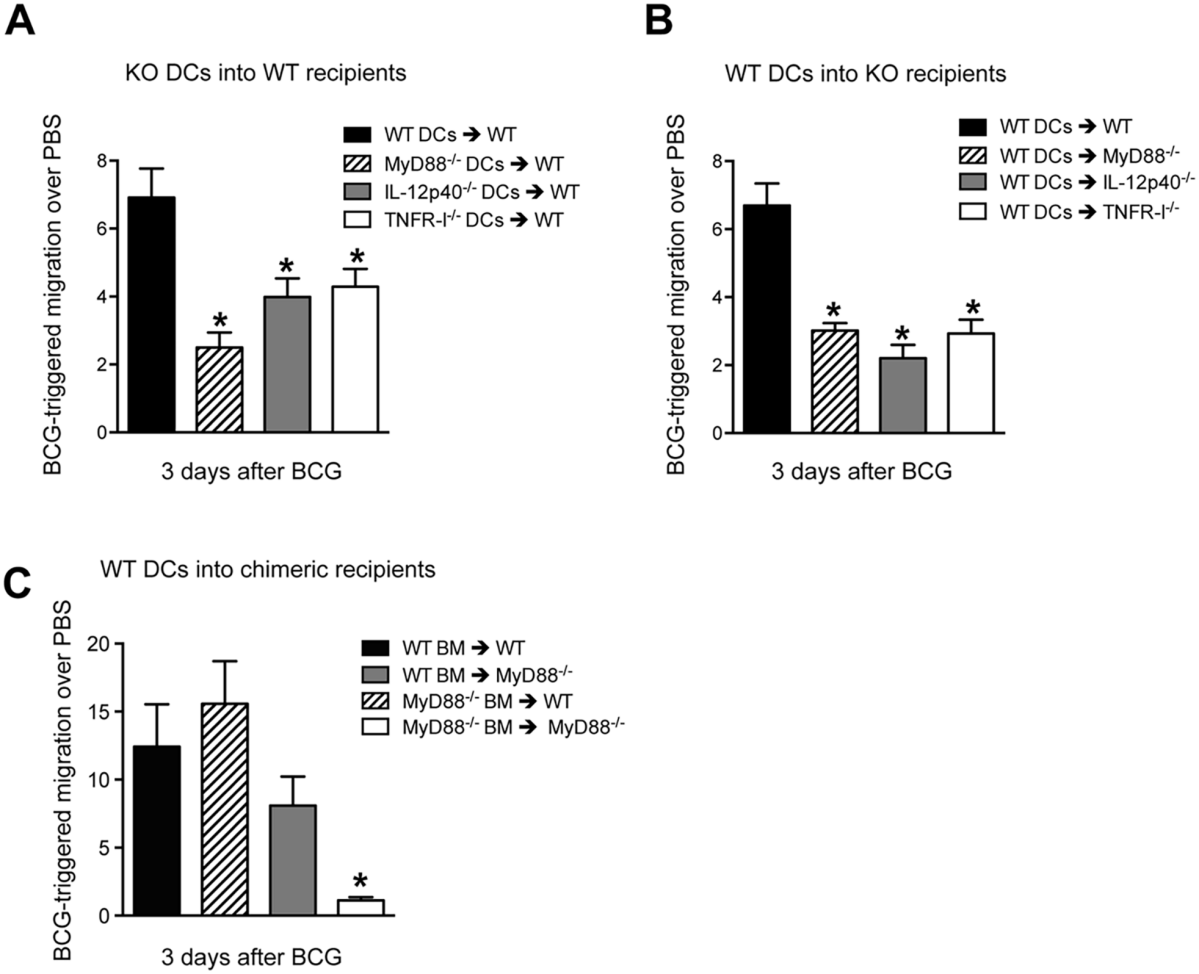
BCG-triggered migration of transferred BMDCs requires DC-intrinsic and -extrinsic expression of MyD88. (**A**) BMDCs were generated from WT, MyD88^-/-^, IL-12p40^-/-^ and TNFR-I^-/-^ mice, labeled with CFSE and injected in the footpad of naïve, WT recipients which were then infected 2hrs later with BCG in the same footpad. The number of labeled BMDCs reaching the draining pLN was determined by flow cytometry 3 days after BCG infection. Migration was expressed as the ratio of DCs that migrated to DLN in response to BCG relative to PBS-injected controls. See S7A Fig for details. (**B**) Reciprocal experiment as in (**A**) where labeled, WT BMDCs were transferred instead into the indicated gene-targeted mice. (**C**) As in (**B**) except labeled WT BMDCs were here transferred into the specified, MyD88^-/-^ bone marrow chimeric recipients. Bars indicate standard error of the mean. Experiments in **A** and **B** each represent pooled data from three separate experiments, each containing 3 to 5 animals per group. *Denotes statistically significant differences between experimental transfers and WT➔WT controls.

The above experiments establish a critical contribution for MyD88 in regulating DC traffic and BCG entry into DLN. We therefore wanted to examine if MyD88 signaling was an intrinsic requirement of the migrating DC. To do so, an adoptive transfer approach with bone marrow-derived DCs (BMDCs) was used where CFSE *in vitro*-labeled BMDCs where injected in the footpad of naïve, recipient mice which where then inoculated 2hrs later with BCG or PBS in the same footpad; the number of transferred DCs arriving in the draining, pLN were then assessed 3 days later by flow cytometry. Although BMDCs tend to migrate poorly in this assay [29], we observed an increased number of transferred DCs in the DLN of BCG-infected animals compared to PBS-injected controls (S7A Fig). To normalize for differences between BMDC culture preparations, we expressed the number of DCs that migrated to the DLN after BCG infection relative to the number of DCs from the same cultures that migrated to the DLN after an injection of PBS (S7A Fig). Using this approach we found that MyD88^-/-^ DCs were muted in their ability to reach the DLN of WT recipients (Fig 7A), suggesting an intrinsic need for MyD88 signaling in the migrating DC. A similar observation was made with IL-12p40^-/-^ and TNFR-I^-/-^ DCs transferred into WT recipients (Fig 7A). Importantly, these findings could not be explained by inherent differences in expression of CCR7, the main chemokine receptor regulating DC entry into LNs [1], as baseline levels of CCR7 mRNA were similar between the various DC cultures investigated (S7B Fig). Indeed, both WT and MyD88^-/-^ BMDCs could migrate across transwells in response to the CCR7 ligand CCL19 (S7C Fig). Interestingly, WT DCs transferred into MyD88^-/-^, IL-12p40^-/-^ or TNFR-I^-/-^ recipients were also unable to migrate efficiently to DLN after BCG footpad infection (Fig 7B). The latter reveals an additional contribution of MyD88 in fueling DC migration where the need for MyD88 is not inherent to the migrating DC itself.

To investigate if this DC-extrinsic requirement for MyD88 was associated to expression of MyD88 in hematopoietic cells, we adoptively transferred WT BMDCs into MyD88^-/-^ bone-marrow radiation chimeric mice. Restoring hematopoietic expression of MyD88 in MyD88^-/-^ mice certainly enhanced BCG-triggered DC migration, implicating the bone marrow in MyD88-dependent DC migration (Fig 7C). On the other hand, rendering the hematopoietic compartment of WT mice deficient in MyD88 did not attenuate BCG-triggered DC migration (Fig 7C). Thus, although MyD88 expression in hematopoietic cells plays an important role in driving migration of adoptively transferred DCs, stromal or radio-resistant cells can at least sustain DC migration in a WT host reconstituted with MyD88^-/-^ bone marrow. These findings reveal an unexpected, interplay between myeloid and stromal cells in MyD88-dependent mobilization of transferred DCs to DLN in response to BCG.

## Discussion

Although DCs play a central role in the initiation of protective Th1 responses to mycobacteria [30], many questions remain unanswered with regards to the priming of CD4^+^ T cells. A central caveat is the transport of mycobacteria from the effector site to the DLN where T-cell responses are initiated. During immunization with model antigens, the magnitude and quality of CD4^+^ T-cell priming is proportional to the number of antigen-bearing DCs reaching the DLN [29]. Indeed, the arrival of increased DC numbers to the DLN can enhance the magnitude of the CD4^+^ T-cell response by favoring the recruitment of multiple T-cell clones and allowing T cells to successively engage multiple DCs [31]. We show in a BCG footpad infection model that migratory EpCAM^low^ CD11b^high^ skin DCs are important for this channeling of antigen by transporting live bacilli to the DLN in an IL-1R-MyD88-dependent manner. The arrival of BCG in the DLN was associated with the priming of mycobacteria-specific P25 TCRTg cells at that site and supports an accumulating body of evidence that live bacilli in the DLN are needed for the initiation of T-cell responses to mycobacteria [32–34].

In spite of similarities, there exist also differences in T-cell priming between BCG and *M*. *tuberculosis*. The priming of CD4^+^ T cells in our BCG model is certainly faster than in *M*. *tuberculosis* [33,35]. The lower bacterial inoculum may delay T-cell activation during *M*. *tuberculosis* [33,35], but is not thought to be the singular determinant of this delay [32]. *M*. *tuberculosis* may initially reside in a static, cellular compartment, which could consequently postpone the transport of bacilli to DLN. Moreover, *M*. *tuberculosis*-infected lung DCs seem to be limited in their capacity to relocate to DLN [35]. On the contrary, our data suggests that transmission of BCG to DLN in skin DCs is rapid and continuous. This provides a possible explanation as to why T-cell priming to BCG is quicker than to *M*. *tuberculosis*.

Our study reveals a role for migratory skin DCs in the priming of CD4^+^ T cells to BCG inoculation, a complex, “antigenic preparation” of microbial debris, secreted microbial products and live bacilli capable of infecting phagocytes. Even after cutaneous injection of a soluble antigen accessible to DCs in the LN via lymphatic drainage, migratory skin DCs that acquire antigen at the injection site are still needed for full-scale priming of CD4^+^ T cells in the DLN [36]. This is likely due to the higher antigenic load found in migratory skin DCs [36]. On the other hand, during infection with Herpes simplex virus (HSV) in the skin, transfer of antigen from migratory to LN-resident DCs is needed for priming CD8^+^ T cells in the DLN [37]. By carrying live bacilli to the DLN, EpCAM^low^ CD11b^high^ migratory skin DCs may promote priming both directly and indirectly. Apposition between DCs and naive P25 TCRTg cells in the T-cell area of the LN provides support for the former scenario. However, it is also possible that DCs that mobilize from the site of antigen deposition to the T-cell area promote priming indirectly by facilitating the transfer of BCG or its antigens to other DC populations in the LN. In this regard, LN-resident, CD8α^+^ DCs can produce copious amounts of IL–12 needed for Th1 differentiation, and excel as an early source of IL-12p40 in BCG-infected spleen [38]. Interestingly, antigen transfer from migratory to LN-resident DCs has been shown to optimize CD4^+^ T-cell priming to *M*. *tuberculosis* in lung DLN [39]. Other, recent studies show that CD11b^+^ LN-resident DCs lining the lymphatic sinus can capture lymph-borne, particulate antigen and prime CD4^+^ T cells, independent of migratory DCs [40]. It is unclear to what degree BCG gains direct access to lymphatics to merit the initiation of T-cell responses via this novel route. Similarly, it remains to be determined whether antigen exchange between DCs in skin DLN also favors the priming of Th1 responses to BCG, or for that matter, if there exists a DC sub-population in the LN that champions the priming of CD4^+^ T cells to mycobacteria. Antigen-presentation assays comparing subsets of migratory skin DCs and LN-resident DCs in priming CD4^+^ T cells will help unravel this. Likewise, assessment of BCG-triggered Th1 responses in transgenic mice where defined DC subsets are absent or can be depleted, as recently investigated during *Candida albicans* infection [41], may unfold their relative contribution to initiating T-cell responses to mycobacteria.

Nevertheless, there is an emerging role for migratory EpCAM^low^ CD11b^high^ skin DCs in immune responses to infection. Corroborating our data, a previous study showed that this subset expands in skin DLN after intradermal infection with BCG and *E*. *coli* [19]. Importantly, this same subset of migratory skin DCs can trigger CD8^+^ T-cell priming to a live, adenoviral vaccine delivered via microneedle arrays [42]. CD11b^high^ (Langerin^neg^ CD103^neg^) skin DCs have also been demonstrated to engulf *Leishmania major* in the dermis [43]. Earlier studies show that submucosal DCs migrate to DLN after intravaginal inoculation of HSV and trigger Th1 responses [20,44]. It is possible that EpCAM^low^ CD11b^high^ skin DCs are at least functionally related to the aforementioned population, or the CD11b^high^ subset of migratory lung DCs that can transport *M*. *tuberculosis* to DLN [35]. Thus, our study together with the above, support a role for EpCAM^low^ CD11b^high^ skin DCs as a migratory cell population capable of relaying antigen to DLN to promote T-cell priming.

The importance of IL-1R-MyD88 signaling in regulating DC migration in our model is in line with the central role for MyD88 in mycobacterial-induced DC activation and the dominant role of IL-1R over TLR signaling in immunity to *M*. *tuberculosis* [45,46]. Our data extend on these findings by showing a role for this pathway in promoting DC and BCG entry into DLN. Interestingly, this contribution was partial, as DC traffic and BCG transport to DLN were not entirely ablated in IL-1R-I^-/-^ and MyD88^-/-^ mice. Additional signaling pathways may thus be involved or come into play in the absence of MyD88. Caspase recruitment domain-containing protein (CARD) 9 could be one such candidate. This adaptor molecule is coupled to several C-type lectin receptors that sense mycobacteria [47] and it has recently been shown to regulate production of IL–1β, TNF-α and IL-12p40 in *M*. *tuberculosis*-infected macrophages [48].

DCs also release IL–1β, TNF-α, and IL-12p40 upon co-culture with mycobacteria, and are in fact the primary source of IL-12p40 in response to *M*. *tuberculosis* and BCG *in vivo* [38]. IL-12p40 and TNFR-I were both necessary in our model for BCG-triggered DC mobilization to DLN. IL-12p40 homodimer has been shown to promote DC migration to DLN in the lungs of *M*. *tuberculosis*- and *Francisella tularensis* LVS-infected mice [26,28] respectively, and to promote the transmigration of *Yersinia pestis*-infected DCs towards CCL19 in transwell assays [27]. Our findings in IL-12p40^-/-^ mice, which are deficient in IL–12, IL–23 and IL-12p40, do not prove that IL-12p40 homodimers regulate DC migration in our model. This is very likely though, given the above studies and the fact that a direct role for IL–12 or IL–23 in DC migration is not well established. Our studies thus build on the idea that IL-12p40 (probably as a p40 homodimer) has an important role in initiating a Th1 response to bacteria that precedes that of *bona fide* IL–12.

It is unclear if an injection of IL-12p40 suffices to mobilize DCs from the skin as previously shown for IL–1β and TNF-α [49]. TNF-α-mediated DC mobilization in particular, is associated to down-regulation of E-cadherin on DCs and increased expression of adhesion molecules and CCL21 on lymphatics [9]. We show that administration of IL-12p40 homodimer can restore BCG-triggered skin DC migration in IL-1R-I^-/-^ mice while treatment with TNF-α at concentrations that suffice to mobilize skin DCs in other models [49] [29] does not. It could thus be that TNF-α acts in our model by inducing IL–1 production rather than as a consequence of IL-1R signaling. Additional studies are needed to clarify this.

Our adoptive transfer experiments show that functional MyD88 signaling is needed not only in the migrating DC itself, but also in the recipient, i.e., extrinsic to the migrating DC. Understanding the impact of the above for T-cell priming is a complex undertaking that merits attention in future investigations aimed at unfolding these mechanisms. In addition, specific depletion of MyD88 on DCs may help clarify DC-intrinsic requirements. In regards to DC migration, it is possible that transferred DCs are already “primed” to migrate as both WT and MyD88^-/-^ BMDCs expressed sufficient levels of CCR7 to sustain migration in response to CCL19. This suggests that BCG infection can play a dual role; on the one hand directly activating the migrating DC and on the other, activating the local effector site to make migration more efficient by other means, e.g. by reactivating the lymphatic endothelium or increasing production of pro-inflammatory cytokines. Indeed, TNF-α can condition a vaccination site and enhance T-cell priming [29]. Another possibility is that the DLN itself may influence the recruitment of DCs from the skin to its microenvironment once the DLN becomes exposed to BCG or its products.

DC transfers in bone-marrow radiation chimeras also reveal a requirement for MyD88 signaling in myeloid cells for relocating the transferred DCs to DLN. This suggests that a migrating DC can receive MyD88-dependent signals for migration, e.g. IL–1, TNF-α and/or IL-12p40, from neighboring myeloid cells. These signals could be derived from other DCs in the skin, neutrophils which home to the skin after BCG infection [15], and/or mast cells, recently shown to regulate skin DC migration [50]. Furthermore, results from our bone marrow chimeras also suggest that stromal cells may play a role in sustaining DC migration. In line, factors derived from lymphatic endothelial cells have recently been shown to at least moderately enhance DC migration in response to inflammatory stimuli [51].

In summary, we identified a migratory skin DC sub-population that relocates to DLN together with BCG in an IL-1R-MyD88-dependent manner and which can engage naïve CD4^+^ T cells during priming. Our findings also implicate myeloid and stromal cells in driving DCs from a BCG effector site to the DLN. These results bare consequences for the quality and magnitude of T-cell priming during infection or cell-based therapy with DCs and as such hold promises for modifying CD4^+^ T-cell responses to BCG, other vaccines of low-to-modest efficacy, and to the implementation of adjunct therapies to infection and tumors.

## Materials and Methods

### Mice

MyD88^-/-^ [52], IL-12p40^-/-^ [53], TNFR-I^-/-^ [54], TLR2^-/-^/TLR9^-/-^ [55], all on a C57BL/6 background, were obtained from the MTC breeding unit, Karolinska Institutet, Stockholm, Sweden. Congenic CD45.1^+^ mice on a C57BL/6 background (B6.SJL/Ptprc^a^) originally from Charles River Laboratories, were also obtained from the MTC breeding unit. IL-1R-I^-/-^ mice [56] on a C57BL/6 background were purchased from the Jackson Laboratory. P25 TCRTg RAG–1^-/-^ mice expressing ECFP were generated by crossing P25 TCRTg RAG–1^-/-^ mice [11] with ECFP mice on a RAG–1^-/-^ background (kindly provided by Dr. Ronald Germain, NIAID, NIH). C57BL/6 mice were used as wild-type (WT) controls. All animals were maintained under specific pathogen-free conditions at the MTC breeding unit. Both male and female mice between 8 and 12 wks old were used.

### Ethics statement

Animal were housed and handled at MTC, Karolinska Institutet, according to the directives and guidelines of the Swedish Board of Agriculture, the Swedish Animal Protection Agency, and Karolinska Institutet (djurskyddslagen 1988:534; djurskyddsförordningen 1988:539; djurskyddsmyndingeheten DFS 2004:4). Experiments were approved by the Stockholm North Animal Ethics Council under the Animal Study Proposals N381/11, N431/12, N397/13 and N171/14.

### Mycobacteria

*Mycobacterium bovis* Bacille Calmette-Guérin (BCG) strain Pasteur (1173P2) and BCG Pasteur expressing the red fluorescent protein DsRed (BCG-RFP, kindly provided by Dr. Nathalie Winter, INRA, Tours, France) [15] were expanded in 7H9 broth as previously described [38]. Quantification of mycobacterial Colony-forming units (CFUs) for bacterial stocks and determination of bacterial load in organs was performed by culture on 7H11 agar supplemented with OADC (Difco).

### Inoculation of mice

Animals were inoculated in the hind footpad with 1x10^6^ CFUs of BCG in 30 μl of PBS. Control animals received an equal volume of PBS. For assessment of cell migration from the footpad skin, animals previously injected with BCG or PBS where injected 24hrs before sacrifice in the same footpad with 20 μl of 0.5 mM 5- and 6-carboxyfluorescein diacetate succinimidyl ester (CFSE) (Invitrogen). In select experiments animals were injected with 50 ng or 300 ng rTNF-α or 2 μg rIL-12p40 homodimer (R&D Systems) in the same footpad that 2hrs earlier was inoculated with BCG or PBS. For assessment of lymphatic drainage to LNs, 20 μl 5% Evan’s blue (Sigma) was injected in the footpad and LNs isolated at different time points after injection. For assessment of CD4^+^ antigen-specific T-cell responses, 1x10^5^ LN cells from P25 TCRTg RAG–1^-/-^ mice were first labeled with 1 μM CFSE and then injected i.v. in the tail vein of congenic CD45.1^+^ recipients in a final volume of 200 μl. For DC adoptive transfer experiments, 2x10^6^ naïve BMDCs generated from WT or transgenic mice were labeled with 3 μM CFSE and then injected into the footpad in a final volume of 40 μl. Two hrs after DC transfer, the same footpads were inoculated with 30 μl PBS or 1x10^6^ CFUs of BCG.

### Generation of single-cell suspensions from tissue

LN, spleen or bone marrow were aseptically removed and processed as previously described [38]. Tissues were either gently homogenized through a 100 micron cell strainer or using a tissue grinder. Homogenates were resuspended in PBS and pelleted at 1200 rpm for 5 min. For spleens, erythrocytes were lysed using ACK lysis buffer unless used for determination of CFUs on 7H11 agar. Resulting single-cell suspensions were washed and counted by Trypan blue exclusion prior to further processing. For quantification of Evan’s blue in LNs, LNs were aseptically removed and incubated for 24hrs in 100 μl formamide solution (Sigma) at 56°C and OD_650nm_ measured on a spectrophotometer (Molecular Devices).

### Flow cytometric staining

Single-cell suspensions from tissues were incubated with various combinations of flourochrome-conjugated rat anti-mouse monoclonal antibodies specific for CD4 (L3T4), CD11b (M1/70), CD11c (HL3), MHC-II I-A/I-E (M5/114.15.2), Ly6G (1A8), CD44 (IM7), CD45.2 (104), CD62L (MEL14), CD69 (H1.2F3), Vβ11 (RR3-15) (BD Biosciences), CD326/EpCAM (G8.8), CD103 (2E7) (Biolegend), CD4 (RM4-5), CD45.1 (A20), CD317/PDCA–1 (927) (eBiosciences), for 45 min in FACS buffer (2% FCS in 5 mM EDTA, 0.1% azide) containing 0.5 mg/ml anti-mouse FcγIII/II receptor (2.4G2) (BD Biosciences). For analysis of intracellular cytokine production, cells were stimulated *ex vivo* for 6hrs with 1 μM Ag85B_240–254_ peptide in the presence of 10 μg/ml Brefeldin A (Sigma). Cells were washed and surface stained with CD4, CD44, CD45.2, CD62L and CD69. Cells were fixed in 2% paraformaldehyde (Electron Microscopy Sciences), permeabilized in 1% saponin (Sigma) and stained with anti-IFN-γ (XMG1.2) (BD Biosciences). Flow cytometry was performed on a LSR-II (BD Biosciences) and analyzed with FlowJo software (Tree Star).

### Confocal microscopy

LNs were excised from naïve and infected animals, fixed overnight with 4% paraformaldehyde/PBS followed by dehydration in 30% sucrose/PBS prior to embedding in Tissue-Tek OCT freezing media (Sakura Finetek). 16 to 20 micron-thick sections were cut on a HM 550 cryostat (Thermo Scientific) and adhered to Superfrost Plus slides (VWR). Sections were permeabilized and blocked in PBS containing 0.3% Triton X–100 (Sigma) and 10% goat serum (Jackson Immunoresearch). This was followed by incubation with AlexaFluor 647-conjugated rat anti-CD3 (17A2) (BD Biosciences). Incubation with unconjugated rat anti-PNAd (MECA–79) (BD Biosciences) was followed by staining with AlexaFlour 647-conjugated secondary antibodies (Invitrogen). Slides were mounted with Prolong Gold (Invitrogen). 3D image stacks of LN sections were acquired on a LSM 780 confocal microscope (Carl Zeiss MicroImaging). Images are displayed as 2D maximum intensity projections using Imaris software (Bitplane).

### Radiation bone marrow chimeras

MyD88^-/-^ or congenic CD45.1^+^ C57BL/6 recipients were sub-lethally irradiated with 2 x 500 Rads and each animal inoculated i.v. with 1 x10^6^ bone marrow cells from MyD88^-/-^ or congenic CD45.1^+^ C57BL/6 mice. Mice were provided with antibiotics in the drinking water for 3 wks and used for experiments 18 to 22 wks after engraftment. Reconstitution was assessed by flow cytometric analysis of blood where no significant differences in engraftment were observed between groups. The frequency of engraftment was approximately 90%.

### Generation of bone marrow-derived Dendritic cells (BMDCs)

BMDCs were generated using recombinant GM-CSF (Biosource) from 6 day-old cultures as previously described [38]. These cells were further enriched for CD11c expression by magnetic selection (Miltenyi) that yielded a population of approximately 98–99% CD11c^+^ cells. Naïve BMDCs were labeled with 3 μM CFSE for adoptive transfer experiments in mice or stimulated overnight at 37°C, 5% CO_2_, with BCG at a multiplicity of infection of 1, for gene expression analysis or transwell migration assays.

### CCR7 gene expression measurement by Realtime PCR

Total RNA was extracted from BMDCs using TRIZOL reagent (Sigma) and reverse transcribed into cDNA using M-MLV reverse transcriptase (Promega). Real-time PCR was performed on an ABI PRISM 7500 sequence detection system (Applied Biosystems) using SYBR Green (Sigma). The relative expression of CCR7 was determined by the 2^(-ΔCT)^ method in which samples were normalized to hypoxanthine phosphoribosyl transferase (HPRT). The following primers pairs were used:

HPRT forward: CCC AGC GTC GTG ATT AGC
HPRT reverse: GGA ATA AAC ACT TTT TCC AAA TCC
CCR7 forward: ACC ATG GAC CCA GGG AAA C
CCR7 reverse: GGT ATT CTC GCC GAT GTA G

### Transwell migration assay

Transwell inserts with an 8 micron pore-size (VWR) were placed on 24-well plates containing 1 μg/ml recombinant CCL19 (Peprotech). Naïve or BCG-stimulated BMDCs (3 x 10^5^) were added to the transwell inserts and incubated at 37°C, 5% CO_2_. Transwells were removed 2hrs later and the number of DCs that reached the bottom of the plate were enumerated by Trypan blue exclusion.

### Statistical analyses

The significance of differences in data group means was analyzed by Student’s *t* test or Anova where appropriate, with a cut-off of p < 0.05. In some experiments, outliers were excluded from analysis following Grubbs’s test for outliers (GraphPad, quickcals).

## Supporting Information

S1 Fig Activation of P25 TCRTg cells in DLN after BCG footpad infection. 1 x10^6^ (**A)** and (**B**) or 1 x10^5^ (**C**) naïve, P25 TCRTg cells were CFSE-labeled and transferred into CD45.1^+^ recipient mice inoculated 24hrs later with BCG in the footpad. The draining, pLN was isolated at different time points after infection and subjected to flow cytometric analysis. **(A)** Dilution of CFSE in P25 TCRTg cells (CD4^+^ CD45.2^+^) at different time points after infection. (**B**) Dot plots showing expression of CD69 relative to CFSE (left panel), and CD44 relative to CFSE (center panel), on gated P25 TCRTg cells 3 days after infection. Expression of CD69, CD62L and CD44 on P25 TCRTg cells that had undergone 2 or more cycles of division compared to transgenic T cells that have not yet diluted CFSE (right panel). (**C**) Total number of P25TCRTg cells at different time points after infection that are CD69^high^ and CD62L^low^ respectively. Bars indicate standard error of the mean. One of two independent experiments shown. Five mice were used for BCG-infected groups and at least 3 for PBS-injected controls. * Denotes statistically significant differences between BCG-infected and PBS-injected groups.
(TIF)

S2 Fig CFSE^+^ migratory skin DCs constitute an activated cell population. WT mice are injected with BCG and CFSE as in Fig 2. On day 3 after infection, pLNs were harvested, homogenized into single-cell suspensions and subjected to flow cytometry where the mean fluorescence intensity (MFI) for CD80 and CD86 was determined on CFSE^+^ and CFSE^neg^ MHC-II^high^ CD11c^+/low^ skin DCs. Five mice were used for BCG-infected groups and 4 for PBS-injected controls. Bars indicate standard error of the mean. * Denotes statistically significant differences between CFSE-positive and -negative skin DCs in BCG- and PBS-injected groups.
(TIF)

S3 Fig Gating strategies for cell populations presented in Fig 2. (TIF)

S4 Fig PTx treatment does not impact the activation profile of transferred P25 TCRTg cells or the viability of BCG. (**A**) Histograms showing changes in MFI for CD69 (left panel) and CD62L (right panel) on transferred P25 TCRTg cells (CD4^+^ CD45.2^+^) from experiment in Fig 5C. (**B**) Data group means from the same analysis graphed. * indicates statistically significant differences between BCG-infected groups and uninfected controls (PBS). (**C**) Congenic CD45.1^+^ recipients were infected with BCG in the footpad and 3 days later inoculated with PTx or PBS in the same footpad. CFSE-labeled, naïve P25 TCRTg cells (were then transferred into these same recipients and the activation profile of transferred P25 TCRTg cells determined 24hrs later by flow cytometry. BCG-infected recipients received either 1x10^5^ or 1x10^6^ P25TCRTg cells while uninfected controls received 1x10^6^ T cells. At least 4 mice were used for BCG-infected groups and 3 for PBS-injected controls. (**D**) Nine aliquots of 10 x10^6^ CFUs of BCG were incubated with 1 μg PTx for 4hrs at 37°C and consequently plated on 7H11 agar for determination of CFUs. Nine additional aliquots were incubated with PBS as a control. Differences between groups are not statistically significant. Bars indicate standard error of the mean.
(TIF)

S5 Fig Early activation profile of naïve P25 TCRTg cells transferred into BCG-infected, gene-targeted mice. Naïve P25 TCRTg cells were CFSE-labeled and transferred i.v. (2 x 10^6^ cells/mouse) into C57BL/6 (WT), MyD88^-/-^, IL-1R-I^-/-^, IL-12p40^-/-^ and TNFR-I^-/-^ recipients. Recipients were then infected with BCG in the footpad and the MFI for CD69 (**A**) and CD62L (**B**) on the surface of transferred P25 TCRTg cells (Vβ11^+^ CFSE^+^) determined by flow cytometry 24hrs later in the DLN. Six animals per group used for BCG-infected cohorts and 3 for uninfected controls. Bars indicate standard error of the mean. * Denotes statistically significant differences between BCG-infected WT and gene-targeted groups.
(TIF)

S6 Fig Local administration of IL-12p40 homodimer but not TNF-α can restore BCG-triggered migration in IL-1R-I^-/-^ mice. Total number of CFSE^+^ MHC-II^high^ CD11c^+/low^ skin DCs in BCG-draining pLN of WT and IL-1R-I^-/-^ 3 days after BCG footpad infection. Two hrs after BCG infection, the same footpads were inoculated respectively with vehicle (0.1% BSA in PBS), 50 ng rTNF-α or 2 μg rIL-12p40 homodimer. Twenty-four hrs before sacrifice, animals were injected with 0.5 mM CFSE in the same footpad. Popliteal LNs were harvested, homogenized into single-cell suspensions and subjected to flow cytometry. Dashed line depicts average number of CFSE^+^ MHC-II^high^ CD11c^+/low^ skin DCs in PBS-injected WT controls receiving vehicle. Bars indicate standard error of the mean. * Denotes statistically significant differences between WT and IL-1R-I^-/-^ mice injected with vehicle, and IL-1R-I^-/-^ mice injected with vehicle vs IL-1R-I^-/-^ mice injected with IL–12(p40)_2_, respectively.
(TIF)

S7 Fig BMDCs in the footpad relocate to the DLN after BCG infection, express CCR7 mRNA and migrate across transwells in response to CCL19. (**A**) Experiment performed as in Fig 7. Naïve, CFSE-labeled WT BMDCs were adoptively transferred into the footpad of WT recipients infected 2hrs later in the same footpad with BCG. Zebra plot showing gating strategy for identification of transferred DCs in draining, pLN 3 days after BCG infection (left panel). Histograms depict expression of CD11b and CD11c on transferred DCs. Number of BMDCs in the pLN of BCG- and PBS-inoculated animals were determined by flow cytometry (graph on left). Number of BMDCs reaching the pLN in response to BCG were divided by the number of BMDCs reaching the pLN in response to PBS and graphed (graph on right). (**B**) BMDCs generated from the indicated gene-targeted mice were left untreated or infected overnight with BCG at a multiplicity of infection (MOI) of 1 and subjected thereafter to RNA extraction and cDNA synthesis. The expression of CCR7 mRNA relative to HPRT was determined by Real-time PCR. RNA samples were pooled from 3 independent experiments. (**C**) BMDCs were generated from the indicated gene-targeted mice, left untreated or infected overnight with BCG (MOI 1) and used in transwell assays as described under Materials and Methods. One of two independent experiments. Bars depict standard error of the mean. * Denotes statistically significant difference relative to PBS-injected controls in (**A**).
(TIF)
