# Supplementary figures and images for: BCG Skin Infection Triggers IL-1R-MyD88-Dependent Migration of EpCAMlow CD11bhigh Skin Dendritic cells to Draining Lymph Node During CD4+ T-Cell Priming
Source: PLoS Pathog. 2015 Oct 6;11(10):e1005206. doi: 10.1371/journal.ppat.1005206 (PMC4594926; doi:10.1371/journal.ppat.1005206)

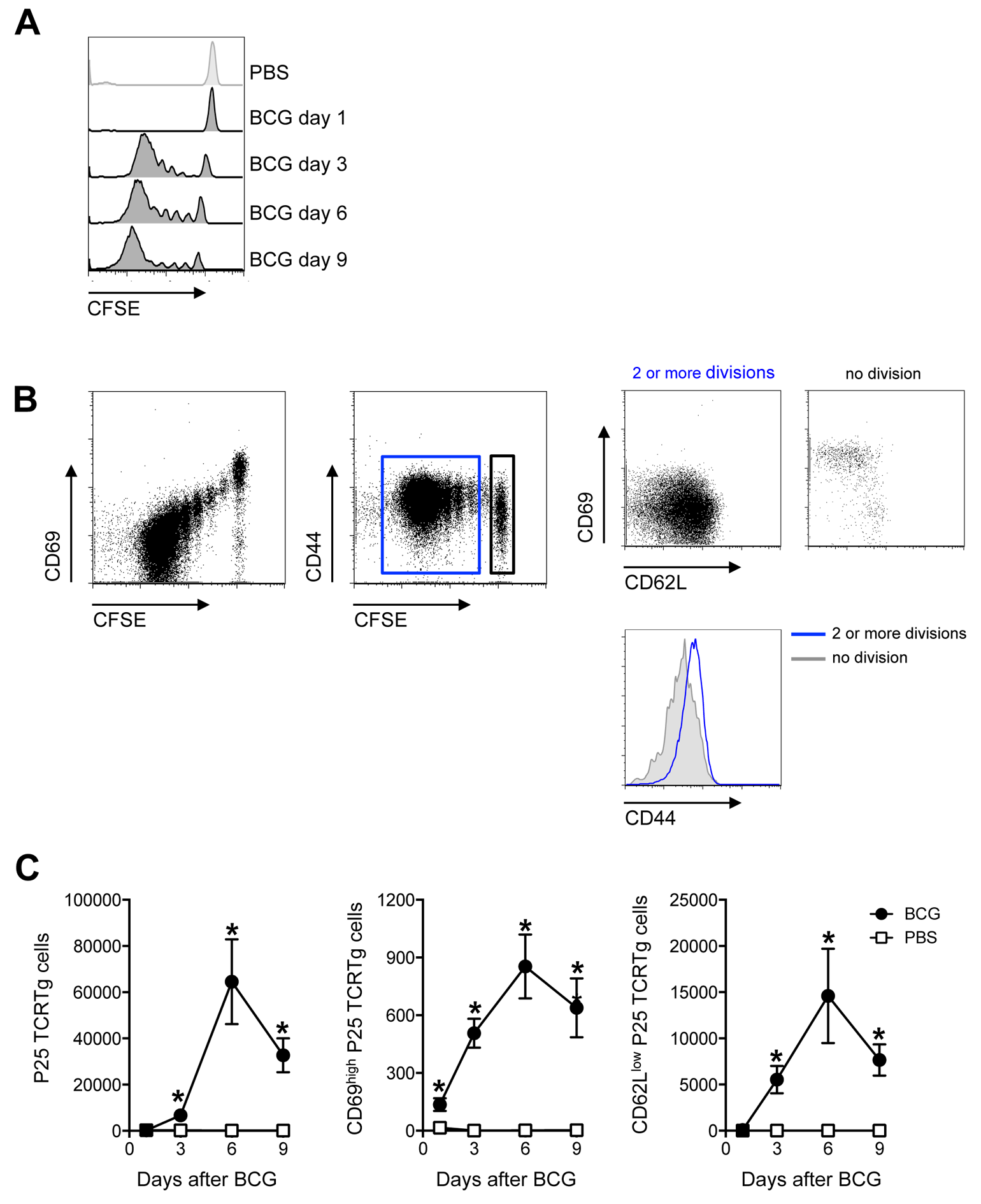

Supplement: S1 Fig — 1 x10^6^ (A) and (B) or 1 x10^5^ (C) naïve, P25 TCRTg cells were CFSE-labeled and transferred into CD45.1+ recipient mice inoculated 24hrs later with BCG in the footpad. The draining, pLN was isolated at different time points after infection and subjected to flow cytometric analysis. (A) Dilution of CFSE in P25 TCRTg cells (CD4+ CD45.2+) at different time points after infection. (B) Dot plots showing expression of CD69 relative to CFSE (left panel), and CD44 relative to CFSE (center panel), on gated P25 TCRTg cells 3 days after infection. Expression of CD69, CD62L and CD44 on P25 TCRTg cells that had undergone 2 or more cycles of division compared to transgenic T cells that have not yet diluted CFSE (right panel). (C) Total number of P25TCRTg cells at different time points after infection that are CD69high and CD62Llow respectively. Bars indicate standard error of the mean. One of two independent experiments shown. Five mice were used for BCG-infected groups and at least 3 for PBS-injected controls. * Denotes statistically significant differences between BCG-infected and PBS-injected groups. (TIF) [file ppat.1005206.s001.tif]

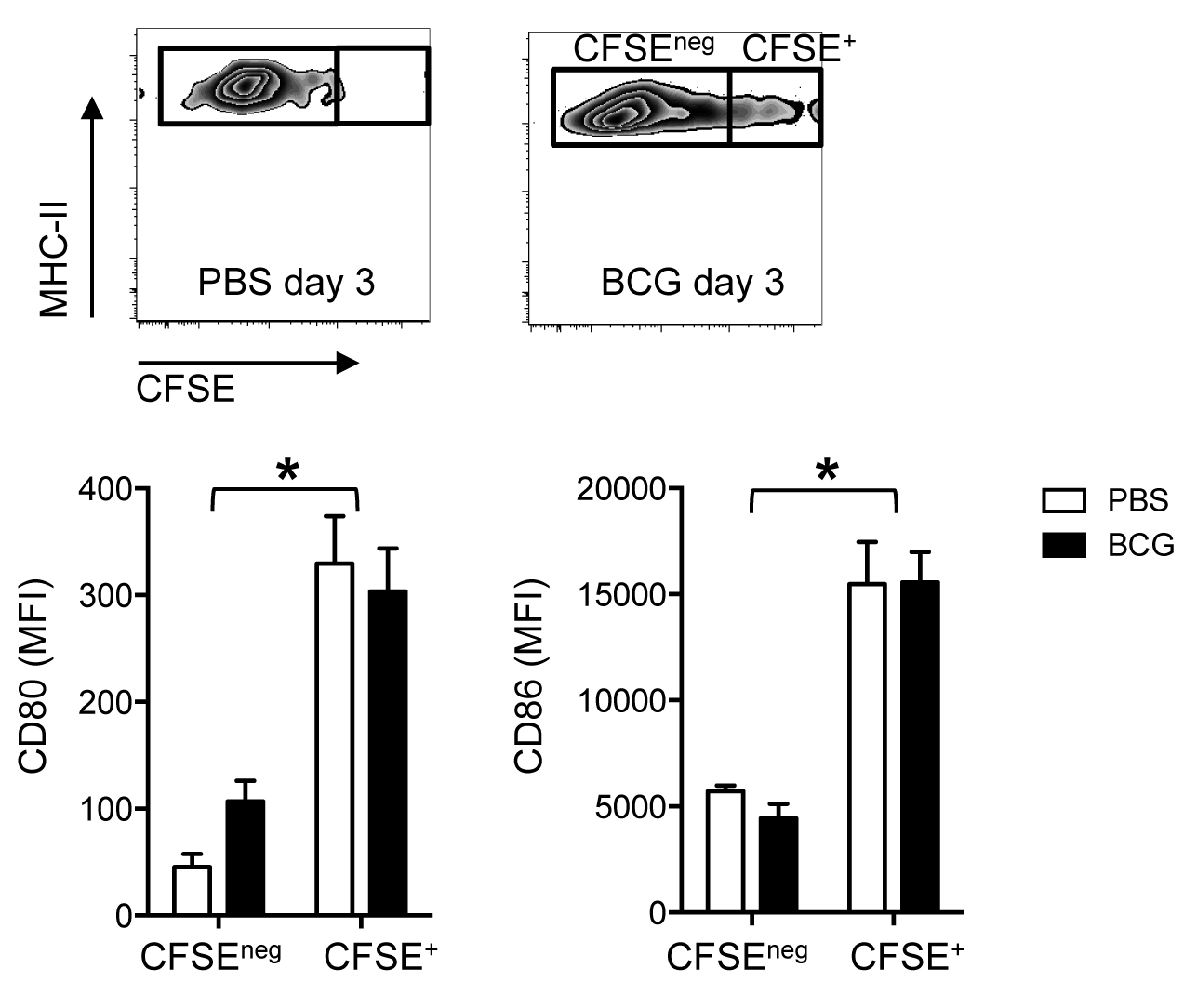

Supplement: S2 Fig — WT mice are injected with BCG and CFSE as in Fig 2. On day 3 after infection, pLNs were harvested, homogenized into single-cell suspensions and subjected to flow cytometry where the mean fluorescence intensity (MFI) for CD80 and CD86 was determined on CFSE+ and CFSEneg MHC-IIhigh CD11c+/low skin DCs. Five mice were used for BCG-infected groups and 4 for PBS-injected controls. Bars indicate standard error of the mean. * Denotes statistically significant differences between CFSE-positive and -negative skin DCs in BCG- and PBS-injected groups. (TIF) [file ppat.1005206.s002.tif]

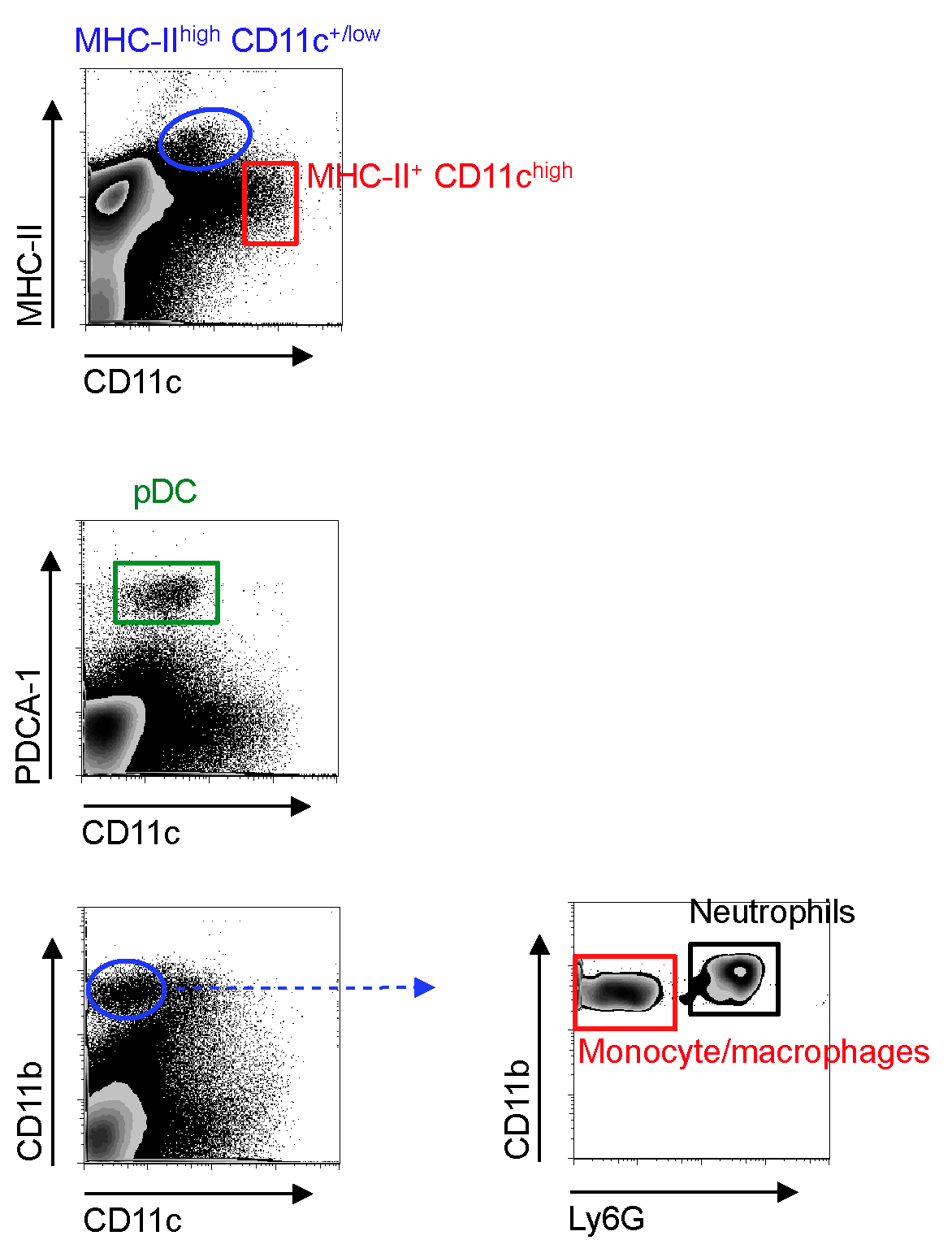

Supplement: S3 Fig — (TIF) [file ppat.1005206.s003.tif]

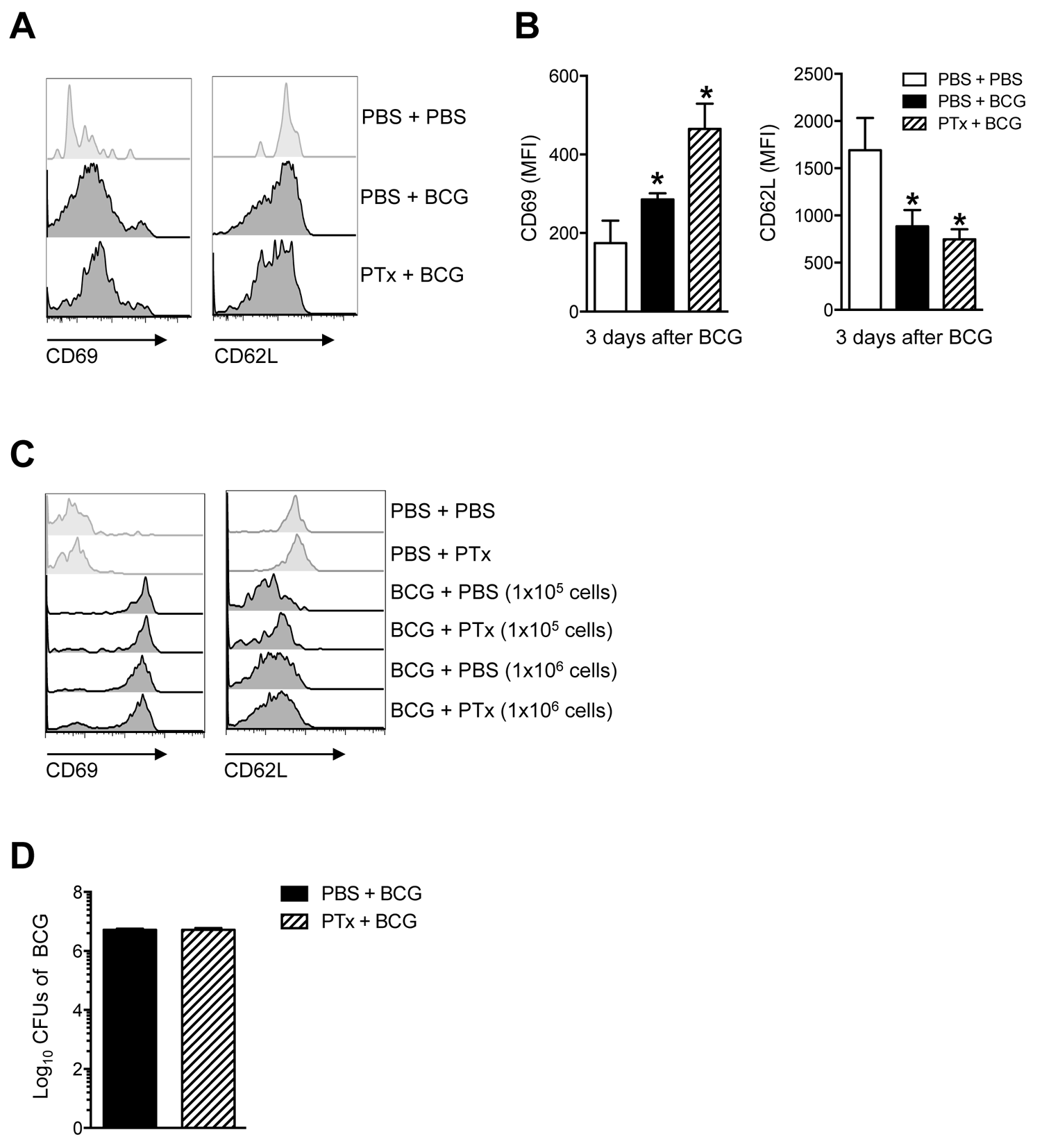

Supplement: S4 Fig — (A) Histograms showing changes in MFI for CD69 (left panel) and CD62L (right panel) on transferred P25 TCRTg cells (CD4+ CD45.2+) from experiment in Fig 5C. (B) Data group means from the same analysis graphed. * indicates statistically significant differences between BCG-infected groups and uninfected controls (PBS). (C) Congenic CD45.1+ recipients were infected with BCG in the footpad and 3 days later inoculated with PTx or PBS in the same footpad. CFSE-labeled, naïve P25 TCRTg cells (were then transferred into these same recipients and the activation profile of transferred P25 TCRTg cells determined 24hrs later by flow cytometry. BCG-infected recipients received either 1x10^5^ or 1x10^6^ P25TCRTg cells while uninfected controls received 1x10^6^ T cells. At least 4 mice were used for BCG-infected groups and 3 for PBS-injected controls. (D) Nine aliquots of 10 x106 CFUs of BCG were incubated with 1 μg PTx for 4hrs at 37°C and consequently plated on 7H11 agar for determination of CFUs. Nine additional aliquots were incubated with PBS as a control. Differences between groups are not statistically significant. Bars indicate standard error of the mean. (TIF) [file ppat.1005206.s004.tif]

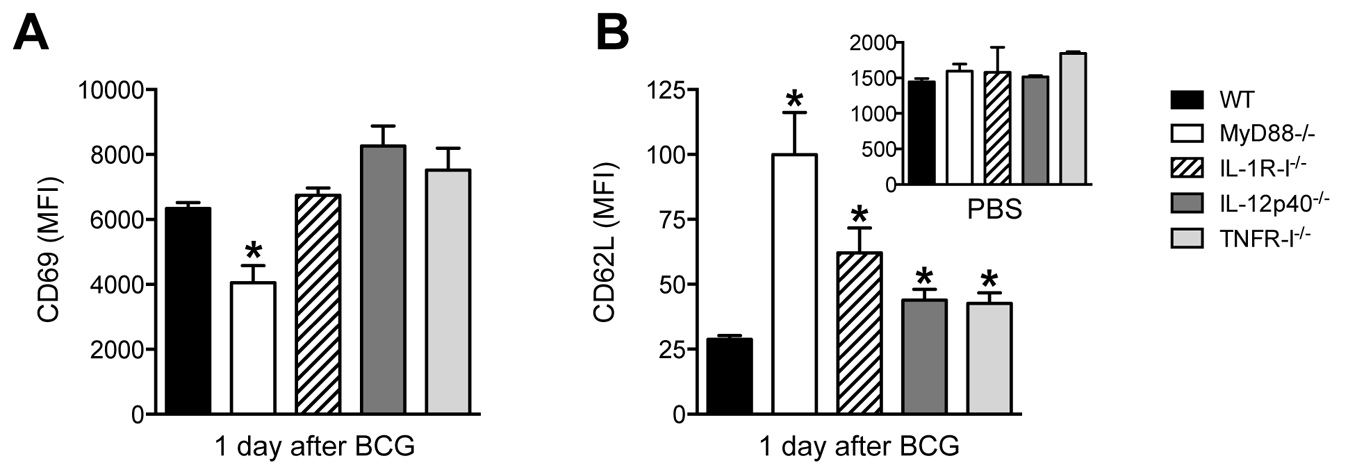

Supplement: S5 Fig — Naïve P25 TCRTg cells were CFSE-labeled and transferred i.v. (2 x 10^6^ cells/mouse) into C57BL/6 (WT), MyD88-/-, IL-1R-I-/-, IL-12p40-/- and TNFR-I-/- recipients. Recipients were then infected with BCG in the footpad and the MFI for CD69 (A) and CD62L (B) on the surface of transferred P25 TCRTg cells (Vβ11+ CFSE+) determined by flow cytometry 24hrs later in the DLN. Six animals per group used for BCG-infected cohorts and 3 for uninfected controls. Bars indicate standard error of the mean. * Denotes statistically significant differences between BCG-infected WT and gene-targeted groups. (TIF) [file ppat.1005206.s005.tif]

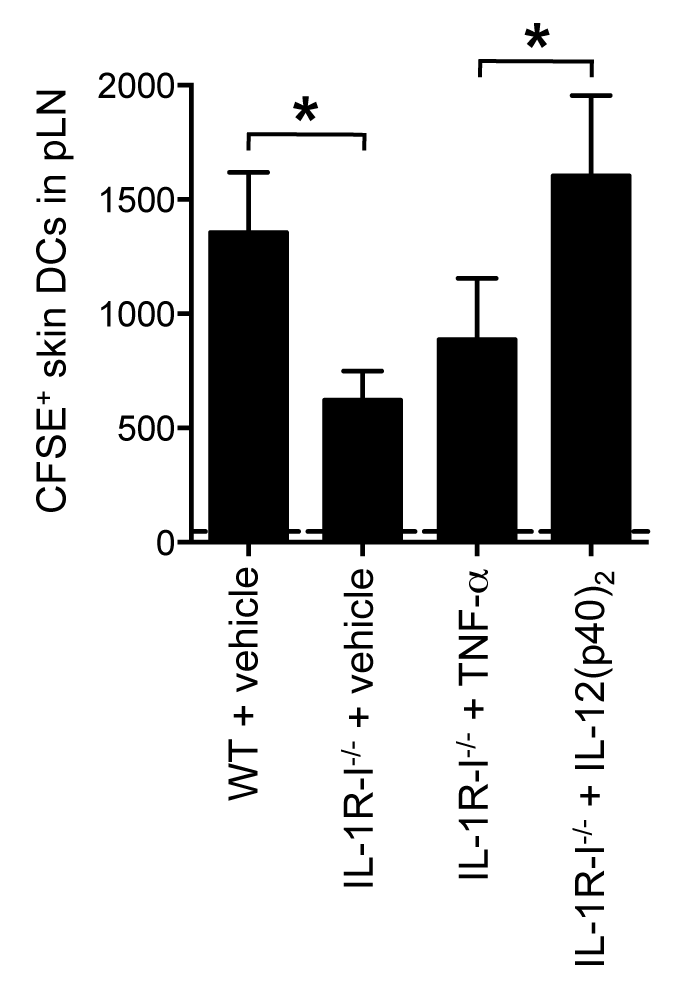

Supplement: S6 Fig — Total number of CFSE+ MHC-IIhigh CD11c+/low skin DCs in BCG-draining pLN of WT and IL-1R-I-/- 3 days after BCG footpad infection. Two hrs after BCG infection, the same footpads were inoculated respectively with vehicle (0.1% BSA in PBS), 50 ng rTNF-α or 2 μg rIL-12p40 homodimer. Twenty-four hrs before sacrifice, animals were injected with 0.5 mM CFSE in the same footpad. Popliteal LNs were harvested, homogenized into single-cell suspensions and subjected to flow cytometry. Dashed line depicts average number of CFSE+ MHC-IIhigh CD11c+/low skin DCs in PBS-injected WT controls receiving vehicle. Bars indicate standard error of the mean. * Denotes statistically significant differences between WT and IL-1R-I-/- mice injected with vehicle, and IL-1R-I-/- mice injected with vehicle vs IL-1R-I-/- mice injected with IL–12(p40)2, respectively. (TIF) [file ppat.1005206.s006.tif]

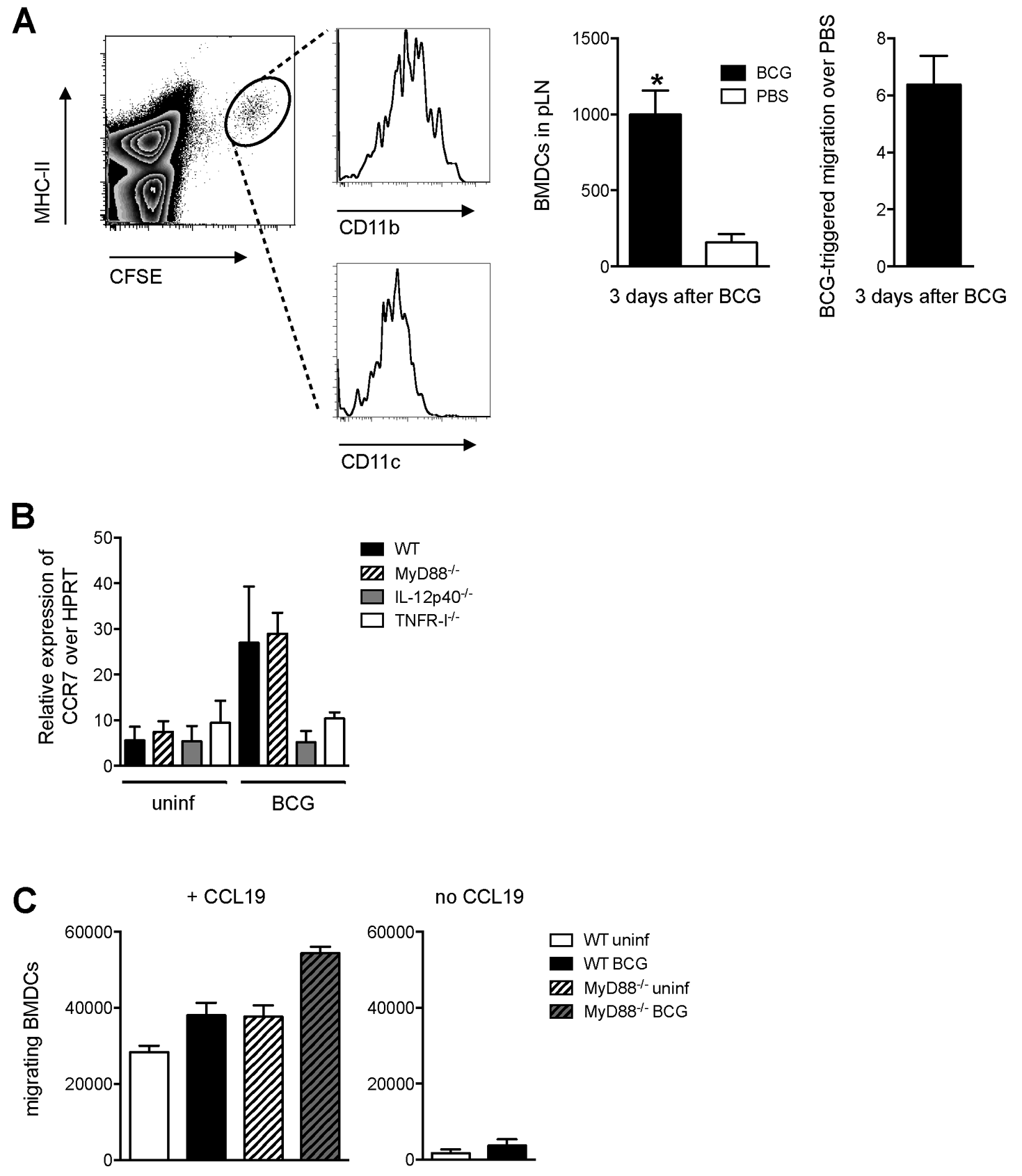

Supplement: S7 Fig — (A) Experiment performed as in Fig 7. Naïve, CFSE-labeled WT BMDCs were adoptively transferred into the footpad of WT recipients infected 2hrs later in the same footpad with BCG. Zebra plot showing gating strategy for identification of transferred DCs in draining, pLN 3 days after BCG infection (left panel). Histograms depict expression of CD11b and CD11c on transferred DCs. Number of BMDCs in the pLN of BCG- and PBS-inoculated animals were determined by flow cytometry (graph on left). Number of BMDCs reaching the pLN in response to BCG were divided by the number of BMDCs reaching the pLN in response to PBS and graphed (graph on right). (B) BMDCs generated from the indicated gene-targeted mice were left untreated or infected overnight with BCG at a multiplicity of infection (MOI) of 1 and subjected thereafter to RNA extraction and cDNA synthesis. The expression of CCR7 mRNA relative to HPRT was determined by Real-time PCR. RNA samples were pooled from 3 independent experiments. (C) BMDCs were generated from the indicated gene-targeted mice, left untreated or infected overnight with BCG (MOI 1) and used in transwell assays as described under Materials and Methods. One of two independent experiments. Bars depict standard error of the mean. * Denotes statistically significant difference relative to PBS-injected controls in (A). (TIF) [file ppat.1005206.s007.tif]
